# Perspectives of Adult Singaporeans toward Potential Policies to Reduce the Consumption of Sugar Sweetened Beverages—A Cross-Sectional Study

**DOI:** 10.3390/nu13124231

**Published:** 2021-11-25

**Authors:** Jing Yuan Tan, Siong Gim Ong, Albert Teng, Benedict Ng, Jiali Yao, Nan Luo, Salome A. Rebello

**Affiliations:** 1 Yong Loo Lin School of Medicine, National University of Singapore, Singapore 119228, Singapore; tanjingyuan72@gmail.com (J.Y.T.); ongsg94@gmail.com (S.G.O.); tengkla94@gmail.com (A.T.); ben4967@yahoo.com.sg (B.N.); 2 Saw Swee Hock School of Public Health, National University of Singapore, Singapore 117549, Singapore; yaojiali@nus.edu.sg (J.Y.); ephln@nus.edu.sg (N.L.)

**Keywords:** food environment, policies, sugar sweetened beverages, attitudes, perceptions, obesity, diabetes

## Abstract

Excessive consumption of sugar sweetened beverages (SSB) is of growing concern, and several countries are implementing measures to reduce SSB consumption. Understanding perceptions towards SSB policies is crucial to prioritize policy actions and to effectively frame public communication. We conducted a cross-sectional study in a sample of 754 adult Singaporeans to examine support towards 10 hypothetical policies to reduce SSB consumption. Policy scenarios were presented to participants and support was assessed using a 5-point Likert scale. Opinions about policies were elicited by asking participants “What other thoughts do you have about this policy?”. We used logistic regression to examine determinants of policy support, and thematic analyses to understand opinions about policies. We observed good public support for a variety of SSB policies. In general, less restrictive policies such as traffic light labels (85.0% agreed/strongly agreed) and free access to water at eateries (77.1%) were better supported as compared to restrictive policies such as portion-size restrictions (64.5%) and taxation (55.0%). There was limited variation by age, ethnicity, income, physical activity and body mass index. Concerns about policies largely centered on loss of personal autonomy and economic implications for businesses. Nevertheless, participants also recognized that policies could support healthier beverage consumption by increasing awareness and enabling informed decision making. Findings from this study provide insights into consumer’s perceptions of SSB policies, and can inform public health advocacy and government action in this area.

## 1. Introduction

Poor dietary behaviors are important contributors to the global burden of disease [1]. Globally 299,521 years of life lost and 8,553,000 years of life lived with disability were attributed to unwise food choices [1]. Amongst dietary behaviors, the excessive consumption of sugar sweetened beverages (SSB) has received considerable attention. In a meta-analyses of prospective cohort studies in adults, each additional serving of SSB was associated with an 18% increase in the risk of type-2 diabetes (T2DM) [2], an 8% increase in the risk of hypertension and a 17% increase in the risk of coronary heart disease [3,4]. Habitual consumption of SSB has also been associated with excessive body weight in children [5].

The rising burden of cardiometabolic disease is predicted to disproportionately affect developing countries, many of which are in Asia. Concurrently, food industries are focusing their attention on emerging markets to expand their business [6]. Developing effective polices to moderate the consumption of sweetened beverages for this region is therefore critical. Several policies to reduce the consumption of SSB have been proposed by international health agencies [7], and are actively being implemented by Asian countries [8]. An important determinant of policy success is public support for the policy [9]. Consumer participation in policy design is thought to provide complementary viewpoints and promote transparency [10]. Surveys conducted in Western settings show more support for less intrusive food policies such as education, as compared to more intrusive policies such as taxation [11]. A recent meta-analysis of nine studies from US, Europe and Australia estimated that 42% of participants supported the SSB tax [12]. Poor public support for fiscal policies has been linked to concerns about the effectiveness of the policy and lack of trust in the government’s use of funds [13]. Consistent with this, earmarking the use of tax-generated revenue for public health programs was associated with improved public support for taxation policies [12,14,15]. Understanding public’s support for, and opinions towards SSB policies is therefore crucial to prioritize policy actions and to craft policy communications that effectively acknowledges and allays legitimate public concerns. However, data on public’s perceptions towards policies aimed to improving dietary behaviors from Asia are lacking [11,12]. Given the differences in socio-cultural, environmental and political landscapes of Asian countries as compared to Western countries, public opinions and concerns may also be different [11].

In Singapore, 36.2% of adults had excess body weight in 2017, and 8.6% had diabetes [16]. The prevalence of diabetes is projected to increase to 15% by 2050 [17], with a total economic cost for working-aged people with diabetes of USD 1867 million [18]. In response, the government announced a war on diabetes in 2016 which included measures to promote healthier eating [19]. Most strategies to limit SSB have typically focused on less restrictive measures such as front of package label for drinks that have a lower sugar content to facilitate easy identification [20]. However, more restrictive policies are actively being considered [21,22] and more recently the government issued a directive which limits the sugar content of drinks available at government premises [23]. The government has also proposed other measures such as mandatory front of pack labeling and additional advertising restrictions for unhealthy drinks [24].

In this study we aimed to examine the level of support of adult Singaporeans for a range of hypothetical policy options intended to reduce the consumption of sugar sweetened beverages (SSB). We also examined demographic, socio-economic, and lifestyle determinants for policy support and assessed their opinions on these policies. Results from this study may help inform the design of policies and the development of effective messaging by public health advocates to reduce SSB consumption in Singapore, and in other countries with similar socio-political contexts.

## 2. Materials and Methods

### 2.1. Study Design and Recruitment

A cross-sectional survey was administered from 7 February 2017 to 12 February 2017 to examine the attitudes and perceptions of adult Singaporeans towards policies aimed at reducing the consumption of SSB. To achieve a geographically representative sample, the country was divided into 5 regions (North, South, East, West and Central) based on the electoral map of the 2015 Singapore general elections. Two constituencies were randomly selected from each of these 5 regions, and 3 housing development board blocks (HDBs) were further randomly selected from each selected constituency for a total of 30 HDB blocks with a total of 3620 apartments. More than 80% of Singaporeans reside in HDBs which are public housing estates managed by the government [25]. All apartments in the selected HDBs were approached to assess eligibility. Participants were eligible if they were Singaporean citizens or permanent residents, aged 21 years and above and able to give verbal informed consent. Only English and Mandarin speakers were included since the questionnaire was administered in these two languages. At each apartment, if a resident responded, they were assessed for eligibility. If eligible, an interview was subsequently administered after verbal consent was obtained. If a resident responded but did not meet the inclusion criteria, he was asked if there were other eligible members in the house. If there were more than one other eligible member in the house, the member whose birthday had most recently passed was selected. Individuals were excluded from the study if they did not fulfil the inclusion criteria or faced significant communication difficulties when the questionnaire was administered. The survey was interview administered and data were electronically captured. The study was approved by National University of Singapore Institutional Review Board (B-16-301).

### 2.2. Questionnaire

The questionnaire included questions assessing participant’s demographics, SSB consumption habits, perceived responsibility of stakeholders in solving obesity, knowledge about T2DM and opinions towards 10 hypothetical policies aimed at reducing consumption of SSB. The policy scenarios were adapted from the World Cancer Research Fund’s NOURISHING framework [8] and from countries who have implemented similar policies [26,27,28,29,30] and were supported by showcards. At the time of this study, the policy scenarios were not implemented in Singapore. Questions assessing participant demographic characteristics and beverage consumption habits were adapted from national surveys [31,32] and a local cohort study [33]. Perceptions related to policy support and stakeholder responsible for solving obesity were adapted from the literature [34,35,36]. Questions were pre-tested to ensure understanding, and were iteratively modified. We also shared the questionnaire with the Singapore Health Promotion Board—a government organization tasked with national health promotion efforts—for feedback. Interviewers were trained in a half-day session, and were provided with standardized scripts. Policy scenarios as presented to the participants can be found in Supplementary Materials Table S1. A copy of the questionnaire is available as supplementary information and show-cards are available on request.

### 2.3. Outcome

The primary outcome of the study was the participant’s level of support towards 10 hypothetical polices aimed at reducing the consumption of SSB. A policy scenario was presented to the participant, with a show card to illustrate the policy. An example of one such scenario was “Consider a policy in which the government will impose a 20% tax on sugar sweetened beverages. For instance, SSB which currently cost consumers SGD 1 will cost consumers SGD 1.20 after this policy is carried out. However, beverages which contain no added sugars will not be taxed.”. Participants were shown the show card and asked to rate their support for the policy on 5-point Likert scale (strongly agree, agree, neither agree nor disagree, disagree, strongly disagree). Using the Nuffield Council on Bioethics’ intervention ladder [37] we broadly classified policies as more restrictive or less restrictive. Restrictive policies included (i) SSB tax (20%), (ii) Restricting sale of SSB in government institutions, (iii) Restricting sale of SSB around schools, (iv) SSB advertisement restriction around schools, and (v) Limiting portion size of SSB. Less restrictive policies included (i) SSB traffic light label, (ii) Warning label, (iii) Installing water fountains at eateries such as food courts and hawker centers, (iv) Warning on SSB marketing materials, and (v) Reducing the visibility of SSB by food vendors at government institutions.

### 2.4. Determinants of Policy Support

We collected data on socio-demographics including age, sex, education level, monthly household income, work status, housing-unit type, having a child 18 years or younger. Data on lifestyle characteristics included self-reported height, and weight, total amount of weekly physical activity and history of chronic medical conditions. Participants were asked about their frequency (daily, weekly, monthly or never/rarely) of consuming sweetened beverages (e.g., Coke, Pepsi, sweetened juices, sweet tea) and non-sweetened beverages (e.g., Diet Coke, non-sweetened juices). We evaluated participants’ perceptions of key stakeholders responsible for solving the problem of obesity in Singapore via a 5-point scale (no/little/moderate/large/very large responsibility). Stakeholders included individuals themselves, family, healthcare professionals, schools, employers and government, and were selected based on the McLeroys’ socio-ecological model for health promotion [38]. Participants were assessed on their knowledge of T2DM using questions adapted from a local study [39]. There were 5 statements on T2DM and participants were asked to select either true, false or unsure for each statement. Participants who responded correctly to at least 3 of these 5 questions were considered as having good diabetes knowledge. We assessed participants’ opinions about each policy by asking them “What other thoughts do you have about this policy.”. Responses were recorded by the interviewers as free text and were subsequently translated if required. Most responses were recorded as phrases and were 10 to 20 words long.

### 2.5. Data Analysis

Participant characteristics were summarized using frequencies (*n*, percentage), mean (95% CI) or median (interquartile range) as appropriate. To examine characteristics of participants who were supportive of most SSB policy scenarios, we compared characteristics across three categories of generic policy support (participants who agreed/strongly agreed to 0–3 policies, 4–6 policies, 7–10 policies) using chi-square tests. Participants’ level of support for a policy was dichotomized to those who supported the policy (participants who indicated that they agree or strongly agree to the statement “I support the policy”) and to those who did not support the policy (participants who neither agreed nor disagreed, disagreed or strongly disagreed). Participants’ perceptions of stakeholders responsible for solving obesity was categorized as having high responsibility (very large responsibility, large responsibility) or moderate/low responsibility (moderate responsibility, low responsibility, no responsibility).

To examine the determinants of policy support we used univariate logistic regression with policy support (yes/no) as the outcome variable, and 3 main groups of determinants, namely socio-demographic characteristics, lifestyle characteristics and opinions as predictor variables. Socio-demographic characteristics included age (21–40 years, 41–64 year or ≥65 years), housing unit (3-room, 4-room, 5-room), monthly household income (less than SGD 4000, SGD 4000–5999, more than SGD 6000), education (primary, secondary, post-secondary, tertiary), work status (not employed, employed, student) and whether the participant had children 18 years and below (yes, no). Lifestyle characteristics included BMI (<23 kg/m^2^, 23–27.5 kg/m^2^, ≥27.5 kg/m^2^) [40], exercise (<150 min/week, ≥150 min/week), having a chronic medical condition specifically hypertension/hyperlipidemia/type 2 diabetes (yes, no) and consumption of SSB (daily, non-daily). Participant perceptions included knowledge that SSB causes health problems (yes, no or unsure), participants’ level of diabetes knowledge (good, poor) and participants opinions towards stakeholders responsible for solving the obesity problem (people themselves, family, school, food industry, healthcare professional, employers, government). Data were analyzed using STATA, and a *p*-value of ≤0.05 was considered statistically significant.

We used thematic analysis to analyze participant open-ended responses to opinions about policies [41]. We chose manual coding as the non-specific nature of the question and the relatively limited number of responses (*n* = 754) made manual coding feasible. A codebook was developed based on an initial reading of the responses for 2 policies, and was subsequently applied to code the responses for other policies. New themes that emerged from subsequent policies were added to the codebook, and were accordingly applied to all responses. Coding was performed by 2 authors (J.Y.T., S.G.O.) and all codes were verified by a third author (S.A.R.). Differences in coding decisions were resolved by discussion. Responses whose meanings was ambiguous (*n* = 25) were coded as undecided and were not included in the analyses.

## 3. Results

### 3.1. Study Recruitment

Out of the 3620 units approached, 1851 units could be contacted of which 956 of those units disagreed to participate in the survey. Of the remaining 895 apartments, respondents from 139 apartments were not eligible. We interviewed one respondent from each apartment from the remaining 756 apartments. Data from 2 respondents were voided due to poor quality of interview leaving a total sample of 754 respondents. The overall response rate was 44.2%. To estimate a prevalence of 50% policy support, with a 99% confidence interval and a precision of 5%, a sample of 664 participants was required. A lower or higher level of policy acceptability would require a smaller sample. For example, to estimate a prevalence of policy support of 75 % with a 99% confidence interval and a precision of 5% a sample of 498 was required.

### 3.2. Socio-Demographic Characteristics

Our participants were mainly Chinese (78.4%) with a mean age of 51.7 years (Table 1). A majority of our participants completed at least secondary school (80.7%) and close to half of our participants were employed (42.5%). About a third of our participants reported having diabetes, hyperlipidemia or hypertension. More than 50% reported consuming sweetened drinks on a daily basis.

### 3.3. Perceptions towards Obesity and SSBs

The vast majority of participants (90.8%) perceived that people themselves have a high responsibility for obesity. Nevertheless, other stakeholders, primarily family (55.3%), school (43.3%) and government (42.8%) were also seen as having a high responsibility. In contrast, only few participants (13.1 %) thought that employers have a high responsibility towards obesity. Most participants (88.6%) recognized that excessive SSB consumption increased the risk of ill health.

### 3.4. Level of Support for SSB Policies

Most people were fairly supportive of SSB polices in general (Table 1) with 43.4% supporting eight policies or more and a majority (92.3%) supportive of at least four policies. Men, those with lower income, less formal education, and those with poor diabetes knowledge were likely to support a fewer number of policies. Levels of policy support were comparable across ethnic groups, work-status categories, SSB consumption levels and BMI categories.

Policy support was generally stronger for less restrictive policy scenarios such as traffic light labels on SSBs (85.0% agree/strongly agree), increasing accessibility to water at eateries (77.1%), and warning labels on SSB packaging (71.9%) (Table 2). Restrictive policies such as taxation (55.0%), SSB portion size restriction (64.5%), and prohibiting sales of high sugar SSBs near schools (65.5%) were less well supported. In contrast, prohibiting the sales of high sugar SSBs at government institutions, a restrictive policy, was supported by a substantial proportion of the participants (74.1%).

### 3.5. Determinants of Policy Support

We observed limited variations in policy support by socio-economic factors such as housing type, education and monthly household income for less restrictive (Table 3, Supplementary Table S2) and restrictive (Table 4, Supplementary Table S3) policies. Policy support was also largely comparable across ethnicities, age-groups, BMI categories, exercise categories and consumption level of SSBs, with some policy-specific exceptions (Table 3 and Table 4). As compared to Chinese, Indians were more likely to support portion-size restrictions for sweetened drinks (OR: 2.33, 95% CI: 1.31–4.14) and restricting sales at government owned institutions (OR: 2.01, 1.06–3.82). Participants who met the minimum physical activity guidelines, were less likely to support policies for SSB health warning labels (0.65, 0.46–0.91), and prohibiting the sale of high-sugar SSB around schools (0.65, 0.47–0.91). As compared to young adults, middle aged adults were more likely to support prohibiting the sale of high-sugar SSB at government institution (1.58, 1.06–2.35), and as compared to those with BMI of <23 kg/m^2^, those with a BMI of 23–27.5 kg.m^2^ were less likely to support the prohibition of sales of high-sugar beverages around schools (0.68, 0.48–0.97).

Women were more likely to support policies related to reducing SSB consumption as compared to men, and were particularly supportive of restricting advertisements around school (OR: 2.48, 95% CI: 1.81–3.39). Participants who reported having a child under the age of 18 years were also more likely to support restrictive school-centric policies including prohibiting the sale of high-sugar SSBs at (1.50, 1.03–2.20) and around (1.51, 1.07–2.13) schools, and prohibiting sweetened beverage advertisements around schools (1.42, 1.00–2.02).

Restrictive policies were more likely to be supported by participants who had good diabetes knowledge. Participants who viewed family members as being important stakeholders in preventing obesity were more supportive of restrictive policies as compared to those who did not consider family members as playing an important role.

### 3.6. Textual Comments

Comments about policy scenarios fitted into four broad categories: comments related to policy effectiveness, comments related to policy ineffectiveness, suggestions for improvements and concerns (Table 5).

#### 3.6.1. Policy Effectiveness

SSB policies were largely seen as an impetus to encourage healthier behaviors. Labeling policies such as the multiple traffic light and warning labels were thought to encourage awareness and increase ease of making the healthier choice. Policies that restricted the sale of high sugar beverages within public sector institutions, and in and around schools were regarded as ways to support the cultivation of healthy habits in the young. Children were considered particularly impressionable, and marketing restriction around schools was also seen as a way of supporting the development of healthy habits. *“Do not feed children with images, children more prone to buy if they see such advertisements”*

Taxation was seen as a deterrent to unhealthy choice as Singaporeans were regarded as being sensitive to price. *“People see money first and then labels”*

Likewise, the provision of free water in local eateries was regarded as providing an economic incentive to drink water rather than sweetened beverages.

#### 3.6.2. Policy Ineffectiveness

Participants viewed personal factors such as individual preferences as an important consideration in food choice behaviors, and this was seen frequently with taxation, and less restrictive polices such as labeling and advertising. Aligned with this, was the perception that sugary drinks are habit-forming, and some people may find it difficult to give this up even with environmental deterrents. *“Nobody cares about the price now, especially when people are addicted.”*

Many comments for policy ineffectiveness were related to specific design features of the policies. For polices related to labels and warnings on advertisements, participants commented that they could be overlooked especially if not prominently displayed. For other less restrictive policies, such as reducing visibility of sweetened drinks, participants thought that this was a weak policy as people could simply request for their beverage of choice.

For restrictive polices such as taxation, several participants commented that a 20% tax is not sufficient to change behavior, particularly for people who crave SSBs. *“Price difference is not as much, people will still pay. Doubling the price then people will feel the pinch.”*

For policies that restrict the sales and advertising around schools, several participants commented that children can still be exposed to SSB advertisements in other settings and can have access to SSBs if they walk a bit more. Others felt that this policy may be difficult to implement as there are many schools in Singapore. Restricting portion size was seen as being ineffective as people could buy more than one serving of the drink.

#### 3.6.3. Concerns

Concerns regarding restrictive SSB policies mainly centered on loss of personal choice. Participants reported that measures such as limiting the sale of high-sugar beverages, or portion sizes were too extreme as they infringed on personal freedom. Another dominant concern which was raised for both restrictive and less restrictive policies was their potential economic impacts. SSB policies such as taxes were seen as increasing the burden on consumers and affecting businesses. *“Things in Singapore already so expensive still want to tax.”*

Policy scenarios which limited the availability or visibility of high sugar drinks were also regarded as being unfriendly to business with some participants remarking on the limited number of low-sugar drink options. *“Don’t really have a large variety of no sugar drinks anyway, then what are they going to put in front sia (Singlish word which denotes emphasis).”*

Concerns for the business of drink vendors was also raised for the policy scenario of installing water fountains in eateries. Participants also cited concerns around hygiene and maintenance for this policy measure. *“Make sure don’t vandalize the cooler or use to wash hands. If too dirty no one will use.”*

#### 3.6.4. Recommendations

Participants emphasized the need for accompanying policy measures with educational campaigns to better prepare and inform consumers. Some participants suggested stronger measures for some policy scenarios, for example not advertising SSBs at all or having more specific information (e.g., exact amount of sugar) for labeling policies. For fiscal measures some participants suggested subsidizing less sweet drinks as a way of encouraging healthier choice.

## 4. Discussion

In this cross-sectional study amongst adult Singaporeans we observed good public support for a variety of hypothetical SSB policies, with limited variation by socio-economic and lifestyle characteristics including age, ethnicity, income, SSB consumption habits, physical activity and BMI. Women were more supportive of policies as compared to men. Participants who had better diabetes knowledge were also more likely to support restrictive policies. In general, less restrictive policies such as labeling and free access to water at eateries were better supported as compared to restrictive policies including taxation and portion-size control. Despite widespread support, participants voiced some concerns about SSB policies, including infringements on personal freedoms, economic implications for industry, and policy effectiveness. However, participants also recognized key merits of SSB policies to support the selection of healthier beverages including increasing awareness and supporting the development of healthier habits in youth.

The higher level of support for less restrictive policies as compared to restrictive policies is consistent with data from Western countries such as the US [35], Germany [42] and Australia [43]. Nevertheless, the level of support we observed for taxation—a policy that was least supported in our population—was still considerably high at 50%. A recent meta-analysis of studies from US, Australia and Europe estimated support for a SSB tax at 42%, (95% CI:  0.38–0.47) [12]. In France, participants were more likely to support SSB tax if the revenue it generated would be used for improving the health-care system or subsidizing healthy foods [14]. Similarly, in New York, a 20% increase in support for taxation was observed if participants were told that the revenue would be used for obesity prevention [15]. Unlike results from other countries [44], concerns around governments’ motivations for implementing the SSB tax or the use of funds thus acquired did not emerge as a major consideration in our study. Rather, some participants were concerned about the economic burden of the tax on the public. Others felt that a 20% tax was not sufficient to encourage behavior change. Price elasticity estimates from the US suggest that a 20% tax, would decrease SSB consumption by 24% [45]. However, comparable data from Singapore are lacking.

Economic implications of SSB polices, primarily on the business of beverage vendors, were also brought up in relation to other policies (e.g., wider access to water), that were supported by most participants. This highlights a perceived tension between policies that could effectively lower SSB consumption, and commercial interests. Conflicting economic pressures that may compromise the implementation of SSB policies have also been highlighted by other stakeholders including academics, politicians, health advocates, and government officials from diverse countries such as Israel [46], New Zealand [47] and Mexico [48]. Potential negative economic implications of SSB polices have been emphasized in media discourses [49], and this may partly shape stakeholders opinions. In our study, participant’s concern was largely expressed for small business such as independently owned drink vendors, rather than for large beverage companies. SSB policy solutions that explicitly incorporate transition plans to help small business adapt, may generate wider public support. Broader efforts to inform the public about the economic costs of obesity and diabetes may also help reframe their perspective [50].

Another area of potential tension was between personal choice and policy effectiveness. Concern with less restrictive policies such as labeling, was that while these regulatory actions may allow people to exercise choice, they may not be fully effective as other factors such as personal preference, apathy or habitual behaviors may play a more dominant role. Similarly, while restrictive policies such as limiting the sale of high-sugar beverages were considered as being valuable measures to limit consumption by some participants, others felt that this intruded on personal freedom. The perception that restrictive policies are intrusive has been previously reported [11,12]. However, modern food environments that are often largely devoid of affordable and appealing heathier options can also be viewed as presenting barriers to personal choice [37,51]. Framing governmental regulations as a mechanism to redress this imbalance may possibly be a constructive way for public health communications to resolve this conflict.

Our observation that women were more in favor of SSB policies, are consistent with data from other countries [11,52]. Additionally, consistent with some other studies [53], we observed that parents of younger children are in favor of efforts to improve the school food environment. In a qualitative study, Singaporean parents with young children voiced mixed opinion about SSB taxation, but were favorably inclined towards other restrictive measures such as ban on sales of higher sugar SSB as this would reduce food-related arguments with their children [54]. These findings suggest that people who are likely to be managing the health of their families [55] may be more sensitized to environmental barriers to healthy living, and thus more appreciative of policies that addresses these barriers. Indeed, we observed that people who viewed family members as playing a major role in addressing issues surrounding obesity, were more in favor of restrictive policies including taxation and portion size restrictions. Other studies have also suggested that participants who recognized that poor food environments play an important role in contributing to obesity may be more likely to support government interventions [42,56]. Improving community understanding of the environmental determinants of health may help improve support for restrictive policies.

Of interest we also noted that people with good knowledge of diabetes were more likely to support restrictive policies. Studies have shown that belief in the harm of risk behaviors such as alcohol consumption, smoking and secondhand e-cigarette vapors were predictors for support for restrictive measures for related policies [57,58,59]. These data suggest that educating consumers not only about the risks associated with high SSB intake but also about the resulting consequences may be important for increasing support for more restrictive policies.

Our results should be interpreted in the light of its limitations. Our sample did not include adults who resided in private housing, or who could not converse in English or Chinese which limits the representativeness of our sample. Inter-interviewer variability was minimized by training interviewers, and providing standardized scripts. Open comments were not available from all participants. The cross-sectional nature of the study precludes conclusions about causality, but is appropriate to assess prevalence of support for various policies.

## 5. Conclusions

This is among the few published studies to examine public support for hypothetical policy scenarios to reduce SSB consumption in Asia, and in Singapore. These findings are particularly timely as the Singapore government is actively considering various strategies to limit consumption of SSB as part of its multi-year war on diabetes. Our results suggest a high public readiness in Singapore for a wide range of strategies to limit SSB consumption. Good public communications to justify the need, and design of the policy may help allay concerns about the policy. Future studies, assessing the perspectives of other relevant stakeholders, such as government, industry including food service establishments and younger participants will be of value.

## Figures and Tables

**Table 1:** Sample characteristics by level of support for policies ^a^.

	**Study Sample**	**Number of Policies Supported**		
	*n* = 754	0–3 Policies	4–7 Policies	8–10 Policies
		*n* = 58	*n* = 369	*n* = 327
**Demographics**				
Gender ^b,c^				
Women	442 (58.6)	16 (3.6)	206 (46.6)	220 (49.8)
Men	312 (41.4)	42 (13.5)	163 (52.2)	107 (34.3)
Age (years) ^b^				
21–40	238 (31.6)	14 (5.9)	137 (57.6)	87 (36.6)
41–64	292 (38.7)	16 (5.5)	147 (50.3)	129 (44.2)
≥65	224 (29.7)	28 (12.5)	85 (37.9)	111 (49.6)
Ethnicity				
Chinese	591 (78.4)	48 (8.1)	289 (48.9)	254 (43.0)
Malay	81 (10.7)	8 (9.9)	42 (51.9)	31 (38.3)
Indian	77 (10.2)	2 (2.6)	36 (46.8)	39 (50.7)
Other	5 (0.7)	0 (0.0)	2 (40.0)	3 (60.0)
Parents with younger children				
No	540 (71.6)	46 (8.5)	262 (48.5)	232 (43.0)
Yes	214 (28.4)	12 (5.61)	107 (50.0)	95 (44.4)
Household monthly income (SGD) ^b^				
<4000	310 (50.4)	30 (9.7)	132 (42.6)	148 (47.7)
4000–5999	146 (23.7)	8 (5.5)	84 (57.5)	54 (37.0)
≥6000	159 (25.9)	9 (5.7)	92 (57.9)	58 (36.5)
Housing unit				
3-room	220 (29.3)	20 (9.1)	101 (45.9)	99 (45.0)
4-room	367 (48.9)	27 (7.4)	178 (48.5)	162 (44.1)
5-room	163 (21.7)	11 (6.8)	89 (54.6)	63 (38.7%)
Work status				
Not employed	378 (50.3)	28 (7.4)	172 (45.5)	178 (47.1)
Employed	319 (42.5)	25 (7.8)	163 (51.1)	131 (41.1)
Student	54 (7.2)	4 (7.4)	33 (61.1)	17 (31.5)
Education ^b^				
Primary	144 (19.3)	18 (12.5)	58 (40.3)	68 (47.2)
Secondary	238 (31.9)	14 (5.9)	103 (43.3)	121 (50.8)
Post-Secondary	135 (18.1)	9 (6.7)	68 (50.4)	58 (43.0)
Tertiary	229 (30.7)	15 (6.6)	138 (60.3)	76 (33.2)
**Lifestyle characteristics**				
Exercise				
<150mins/week	537 (71.2)	44 (8.2)	252 (46.9)	241 (44.9)
≥150mins/week	217 (28.8)	14 (6.5)	117 (53.9)	86 (39.6)
Chronic medical conditions				
No	495 (65.6)	43 (8.7)	246 (49.7)	206 (41.6)
Yes	259 (34.4)	15 (5.8)	123 (47.5)	121 (46.7)
BMI (kg/m^2^)				
<23	374 (53.6)	21 (5.6)	181 (48.4)	172 (46.0)
23−27.5	221 (31.7)	21 (9.5)	113 (51.1)	87 (39.4)
≥27.5	103 (14.8)	8 (7.8)	46 (44.7)	49 (47.6)
Consumption of SSB				
Never or rarely	103 (13.7)	8 (7.8)	49 (47.6)	46 (44.7)
≥1 per month but <1 per week	59 (7.8)	1 (1.7)	24 (40.7)	34 (57.6)
≥1 per week but <1 per day	202 (26.8)	17 (8.4)	103 (51.0)	82 (40.6)
1 or more per day	390 (51.7)	32 (8.2)	193 (49.5)	165 (42.3)
Any policy comments ^b^				
No	360 (47.7)	25 (6.9)	159 (44.2)	176 (48.9)
Yes	394 (52.3)	33 (8.4)	210 (53.3)	151 (38.3)
**Knowledge and attitudes**				
Diabetes knowledge ^b^				
Poor	237 (31.4)	28 (11.8)	122 (51.5)	87 (36.7)
Good	517 (68.6)	30 (5.8)	247 (47.8)	240 (46.4)
SSB causes health problems				
No/unsure	86 (11.4)	12 (14.0)	38 (44.2)	36 (41.9)
Yes	668 (88.6)	46 (6.9)	331 (49.6)	291 (43.6)
Perceived responsibility of stakeholders for solving obesity				
People themselves ^b^				
*High responsibility*	685 (90.8)	46 (6.7)	340 (49.6)	299 (43.7)
*Low-moderate responsibility*	69 (9.2)	12 (17.4)	29 (42.0)	28 (40.6)
Family ^b^				
*High responsibility*	417 (55.3)	26 (6.2)	191 (45.8)	200 (48.0)
*Low-moderate responsibility*	337 (44.7)	32 (9.5)	178 (52.8)	127 (37.7)
Health care professionals ^b^				
*High responsibility*	252 (33.4)	11 (4.4)	120 (47.6)	121 (48.0)
*Low-moderate responsibility*	502 (66.6)	47 (9.4)	249 (49.6)	206 (41.0)
Food industry				
*High responsibility*	304 (40.3)	19 (6.3)	147 (48.4)	138(45.4)
*Low-moderate responsibility*	450 (59.7)	39 (8.7)	222 (49.3)	189 (42.0)
School				
*High responsibility*	327(43.4)	19 (5.8)	147 (45.0)	161 (49.2)
*Low-moderate responsibility*	427 (56.6)	39 (9.1)	222 (52.0)	166 (38.9)
Government policies				
*High responsibility*	323 (42.8)	18 (5.6)	156 (48.3)	149 (46.1)
*Low-moderate responsibility*	431 (57.2)	40 (9.3)	213 (49.4)	178 (41.3)
Employers				
*High responsibility*	99 (13.1)	5 (5.1)	48(48.5)	46 (46.5)
*Low-moderate responsibility*	655 (86.9)	53 (8.1)	321 (49.0)	281 (42.9)

**^a^** Counts may not always add up to 754 due to missing data. ^b^ Significant at *p* ≤ 0.05 based on chi-square test. ^c^ Frequencies (percent) all such numbers.

**Table 2:** Levels of policy support for 10 hypothetical policies (%).

		Overall Support ^a^	Strongly Agree	Agree	Neither Agree nor Disagree	Disagree	Strongly Disagree
**Less** **Restrictive Policies**	Product labeling						
	Traffic light labels	85.0	45.6	39.4	6.1	6.8	2.1
	Warning labels	71.9	26.9	45.0	7.3	17.8	3.1
	Marketing						
	Safety warning on SSB marketing	66.8	22.8	44.0	11.0	19.0	3.2
	Built environment						
	Installing water fountains at eateries	77.1	42.6	34.5	5.8	14.3	2.8
	Choice architecture						
	Reduced visibility of SSB at government owned institutions	60.3	17.5	42.8	12.2	22.3	5.2
**More** **Restrictive Policies**	Taxation						
	SSB tax	55.0	21.0	34.1	9.5	28.2	7.2
	Restrictions						
	Product availability at government-institutions	74.1	33.7	40.5	9.5	13.7	2.7
	Product availability near schools	65.5	23.7	41.8	12.6	18.2	3.7
	Advertising near schools	68.0	20.6	47.5	14.2	15.3	2.5
	Portion size	64.5	20.6	43.9	9.2	21.9	4.5

^a^ Overall support is the sum of people who agree and strongly agree with the policy scenario; SSB: sugar-sweetened beverages.

**Table 3:** Determinants of support for less restrictive policies, odds ratios (95% CI) ^a^.

	Product Labeling		Built Environment	Marketing	Choice Architecture
	Traffic Light Labeling	Warning Labels	Installing Water Fountains at Eateries	Safety Warning on SSB Marketing	Reduced Visibility of SSB at Government-Owned Institutions
**Gender**					
Male	1	1	1	1	1
Female	1.98 * (1.32–2.97)	1.92 * (1.39–2.64)	1.42 * (1.01–1.99)	1.30 (0.95–1.76)	1.95 * (1.45–2.63)
**Work status**					
Not employed	1	1	1	1	1
Employed	1.05 (0.69–1.59)	0.69 (0.50–0.97)	0.93 (0.65–1.32)	1.05 (0.76–1.44)	0.96 (0.71–1.31)
Student	1.04 (0.47–2.32)	1.17 (0.59–2.32)	1.30 (0.63–2.68)	1.01 (0.55–1.85)	0.80 (0.45–1.41)
Exercise					
<150min/week	1	1	1	1	1
≥150min/week	1.03 (0.66–1.60)	0.65* (0.46–0.91)	1.15 (0.79–1.69)	0.97 (0.69–1.35)	0.85 (0.62–1.17)
**Chronic medical conditions**					
No	1	1	1	1	1
Yes	1.32 (0.85–2.04)	1.62* (1.14–2.29)	1.01 (0.71–1.45)	1.08 (0.78–1.49)	1.12 (0.82–1.53)
**Knowledge and Perceptions**					
SSB cause health problems					
No/Unsure	1	1	1	1	1
Yes	1.35 (0.75–2.41)	0.99 (0.60–1.63)	1.44 (0.87–2.37)	1.29 (0.81–2.06)	2.34 * (1.48–3.69)
Diabetes mellitus knowledge					
Poor knowledge	1	1	1	1	1
Good knowledge	1.18 (0.77–1.80)	1.36 (0.97–1.90)	1.34 (0.94–1.91)	1.13 (0.82–1.56)	1.50 * (1.10–2.05)
**Perceived responsibility for solving obesity ^b^**					
People themselves	2.39 * (1.35–4.23)	1.74* (1.04–2.90)	1.53 (0.89–2.64)	1.42 (0.86–2.36)	1.35 (0.82– 2.23)
Family members	1.62 * (1.08–2.42)	1.18 (0.86–1.62)	1.22 (0.87–1.72)	1.34 (0.99–1.82)	1.29 (0.96–1.73)
Health care professionals	1.20 (0.78–1.85)	1.58* (1.11–2.24)	1.14 (0.79–1.64)	1.13 (0.82–1.56)	1.28 (0.94–1.76)
Food industry	1.28 (0.84–1.94)	0.93 (0.68–1.29)	1.20 (0.84–1.70)	1.42 * (1.03–1.94)	1.31 (0.97–1.76)
School	1.48 (0.98–2.24)	0.92 (0.67–1.27)	1.10 (0.78–1.55)	1.39 * (1.02–1.90)	1.50 * (1.11–2.01)
Government policies	1.38 (0.91–2.09)	1.05 (0.76–1.45)	1.46 * (1.03–2.07)	1.19 (0.87–1.62)	1.12 (0.84–1.51)
Employers	1.19 (0.64–2.22)	0.94 (0.59–1.49)	1.12 (0.67–1.88)	1.87 * (1.13– 3.08)	1.44 (0.92–2.26)

BMI = body mass index; CI = confidence interval; SSB = sugar-sweetened beverages * *p*-value<0.05 based on univariate logistic regression models. ^a^ The table only shows participant characteristics that were statistically significantly different or where 95% CI of estimates do not include 1. All sample characteristics are shown in Supplementary Table S2; ^b^ Reference group comprises of participants who consider the stakeholder as having low-moderate responsibility in solving obesity.

**Table 4:** Determinants of support for more restrictive policies, odds ratios (95% CI) ^a^.

	Taxation	Restrictions			
	SSB Tax (20%)	Product Availability at Government-Owned Institutes	Product Availability Near Schools	Advertising Near Schools	Portion Sizes
Age (years)					
21–40	1	1	1	1	1
41–64	0.91 (0.64–1.28)	1.58 * (1.06–2.35)	0.92 (0.64–1.32)	1.09 (0.76–1.58)	1.15 (0.81–1.64)
≥65	1.02 (0.70–1.47)	1.04 (0.70–1.56)	1.11 (0.75–1.63)	1.01 (0.68–1.49)	1.16 (0.79–1.70)
Gender					
Male	1	1	1	1	1
Female	1.35 * (1.01–1.81)	1.54 * (1.11–2.14)	1.59 * (1.18–2.16)	2.48 * (1.81–3.39)	1.69 * (1.25–2.29)
Ethnicity					
Chinese	1	1	1	1	1
Malay	1.11 (0.69–1.77)	1.06 (0.62–1.80)	0.88 (0.55–1.42)	0.67 (0.42–1.08)	1.16 (0.71–1.88)
Indian	1.06 (0.66–1.72)	2.01 (1.06–3.82)	1.67 (0.97–2.88)	1.15 (0.68–1.94)	2.33 * (1.31–4.14)
Other ^b^	-	0.56 (0.09–3.30)	2.19 (0.24–19.71)	-	-
Have children ≤18 years old					
No	1	1	1	1	1
Yes	1.01 (0.73–1.38)	1.50 * (1.02–2.20)	1.51 * (1.07–2.13)	1.42 * (1.00–2.02)	0.92 (0.66–1.28)
Education					
Primary	1	1	1	1	1
Secondary	1.53 (1.01–2.32)	0.90 (0.55–1.45)	1.13 (0.73–1.76)	1.06 (0.68–1.66)	1.44* (0.93–2.23)
Post-secondary	1.05 (0.66–1.68)	1.04 (0.59–1.81)	0.85 (0.52–1.39)	1.00 (0.61–1.66)	1.24 (0.76–2.03)
Tertiary	1.02 (0.67–1.55)	0.73 (0.45–1.18)	0.85 (0.55–1.31)	0.93 (0.59–1.45)	0.85 (0.55–1.30)
Work status					
Not employed	1	1	1	1	1
Employed	1.07 (0.80–1.45)	0.74 (0.52–1.04)	0.82 (0.59–1.12)	0.90 (0.65–1.24)	0.70 (0.51–0.95)
Student	0.56 (0.31–0.99)	0.45 * (0.25–0.82)	0.57 (0.32–1.02)	0.49 (0.27–0.87)	0.66 (0.37–1.18)
BMI (kg/m^2^)					
<23	1	1	1	1	1
23–27.5	0.73 (0.52–1.02)	1.01 (0.69–1.48)	0.68 * (0.48–0.95)	0.86 (0.60–1.23)	0.98 (0.69–1.40)
≥27.5	1.04 (0.67–1.62)	1.49 (0.87 –2.56)	1.16 (0.72–1.88)	1.10 (0.68–1.79)	0.98 (0.62–1.55)
Exercise					
<150min/week	1	1	1	1	1
≥150min/week	1.01 (0.74–1.39)	1.04 (0.72–1.49)	0.65 * (0.47–0.91)	1.07 (0.76–1.51)	1.03 (0.74–1.44)
Diabetes mellitus knowledge					
Poor knowledge	1	1	1	1	1
Good knowledge	1.20 (0.88–1.64)	1.73 * (1.23–2.43)	1.50 * (1.09–2.07)	1.75 * (1.27–2.41)	1.60 * (1.16–2.19)
Perceived responsibility for solving obesity ^c^					
People themselves	1.21 (0.74–1.99)	0.93 (0.52–1. 65)	1.09 (0.65–1. 82)	1.00 (0.59–1. 69)	1.27 (0.76–2.10)
Family members	1.34 * (1.00–1.79)	1.35 (0.98–1. 88)	1.35 * (1.00–1. 83)	1.17 (0.86– 1. 59)	1.57 * (1.16–2.12)
Health care professionals	1.06 (0.78–1.43)	1.34 (0.94–1. 91)	1.14 (0.83–1. 57)	1.46 * (1.05–2.05)	1.43 * (1.04–1. 98)
Food industry	1.06 (0.79–1.42)	1.14 (0.82–1. 60)	1.05 (0.77–1. 42)	1.11 (0.81–1. 52)	1.41 * (1.03–1. 92)
School	1.14 (0.85– 1.52)	1.43 * (1.02–2.00)	1.20 (0.89–1. 63)	1.40 * (1.03–1. 92)	1.19 (0.88–1. 61)
Government policies	1.03 (0.77–1. 37)	1.11 (0.79–1. 54)	1.25 (0.92–1. 70)	1.06 (0.78–1. 44)	1.15 (0.85–1. 55)
Employers	0.98 (0.64–1. 49)	1.34 (0.81–2.24)	2.00 * (1.21–3.29)	1.55 (0.95–2. 52)	1.06 (0.68–1. 66)

BMI = body mass index; CI = confidence interval; SSB = sugar-sweetened beverages, * *p*-value<0.05 based on univariate logistic regression models. ^a^ The table only shows participant characteristics that were statistically significantly different or where 95% CI of estimates do not include 1. All sample characteristics are shown in Supplementary Table S3; ^b^ Estimates were not generated due to low numbers (-); ^c^ Reference group comprises of participants who consider the stakeholder as having low-moderate responsibility in solving obesity.

**Table 5:** Themes related to comments about SSB policies ^a^.

	Less Restrictive Policies					More restrictive Policies				
	Traffic Light Labeling	Warning Label	Safety Warning on SSB Marketing Materials	Installing Water Fountains at Eateries	Reduce Visibility of SSB at Government-Owned Institutions	SSB Tax (20%)	Restricting Sale of SSB at Government-Owned Institutions	Restricting Sale of SSB Near Schools	SSB Advertisement Restriction Near Schools	Limiting Portion Size of SSB
*n*	150	137	105	200	122	171	130	126	64	99
**Effective**										
Encourages healthy behavior	42 (28.0)	11 (8.0)	9 (8.6)	38 (19.0)	16 (13.1)	15 (8.8)	30 (23.1)	20 (15.9)	6 (9.4)	9 (9.1)
Targeted effectiveness	6 (4.0)	9 (6.6)	3 (2.9)	1 (0.5)	-	4 (2.3)	6 (4.6)	5 (4.0)	-	2 (2.0)
Address root of problem	-	-	-	-	-	-	-	5 (4.0)	12 (18.8)	-
Environmental benefits	-	-	-	-	-	-	-	-	-	3 (3.0)
**Ineffective**										
Personal factors	42 (28.0)	33 (24.1)	27 (25.7)	20 (10.0)	24 (19.7)	44 (25.7)	5 (3.8)	3 (2.4)	3 (4.7)	3 (3.0)
Policy design	20 (13.3)	54 (39.4)	26 (24.8)	3 (1.5)	38 (31.1)	61 (35.7)	24 (18.5)	40 (31.7)	18 (28.1)	50 (50.5)
Distrust of information	11 (7.3)	2 (1.5)	-	-	-	2 (1.2)	2 (1.5)	-	-	-
**Concerns**										
Personal rights	-	4 (2.9)	3 (2.9)	-	11 (9.0)	2 (1.2)	32 (24.6)	17 (13.5)	3 (4.7)	20 (20.2)
Economic impact	3 (2.0)	-	7 (6.7)	36 (18.0)	22 (18.0)	22 (12.9)	8 (6.2)	20 (15.9)	6 (9.4)	9 (9.1)
Administrative challenges	4 (2.7)	2 (1.5)	-	7 (3.5)	-	1 (0.6)	-	-	-	-
Nutritional requirements	-	-	-	-	1 (0.8)	-	8 (6.2)	2 (1.6)	-	-
Reduced efficacy	-	-	3 (2.9)	-	-	8 (4.7)	4 (3.1)	-	-	-
Health impact	-	-	-	71 (35.5)	-	-	-	-	-	-
Social impact	-	-	-	2 (1.0)	-	-	-	-	-	-
**Improve implementation**										
Alternative policies	-	-	-	-	5 (4.1)	-	2 (1.5)	5 (4.0)	6 (9.4)	2 (2.0)
Require supporting campaigns	10 (6.7)	6 (4.4)	9 (8.6)	3 (1.5)	3 (2.5)	5 (2.9)	6 (4.6)	5 (4.0)	6 (9.4)	-
Presentation	11 (7.3)	16 (11.7)	5 (4.8)	-		-	-	-	-	-
Policy design	-	-	8 (7.6)	-	2 (1.6)	-	-	-	-	-
**Others**	1 (0.7)	-	-	-	-	-	-	-	2 (3.1)	-
**Undefined**	-	-	5 (4.8)	9 (4.5)	-	7 (4.1)	3 (2.3)	4 (3.2)	2 (3.1)	-

^a^ Numbers are *n* (%); dashes indicate that this theme was not brought up by the participants in relation to this policy.

## Data Availability

Show-cards are available on request from the corresponding author.

## Supplementary Materials

The following are available online at https://www.mdpi.com/article/10.3390/nu13124231/s1, Questionnaire, Table S1: Description of policy scenarios presented to participants, Table S2: Determinants of support for less restrictive policies, odds ratios (95% CI), Table S3: Determinants of support for more restrictive policies, odds ratios (95% CI).

## Abbreviations

BMI, body mass index; HDB; housing development board; SSB, sugar sweetened beverage; STATA, software for statistics and data science; T2DM, type 2 diabetes mellitus.
