# Supplementary material for: Perspectives of Adult Singaporeans toward Potential Policies to Reduce the Consumption of Sugar Sweetened Beverages—A Cross-Sectional Study
Source: Nutrients. 2021 Nov 25;13(12):4231. doi: 10.3390/nu13124231 (PMC8706143; doi:10.3390/nu13124231)
Supplement: Supplementary file 1 [file nutrients-13-04231-s001.zip › nutrients-1360538-supplementary/Supplementary Files/Questionnaire.pdf]

Appendix 1A – English Questionnaire

DATE: __ __/ __ __/__ __/ **START TIME: _______** **END TIME: _________**
(INTERVIEWER: Please use the format of DD/MM/YY)
(INTERVIEWER: Please indicate if AM or PM)

INTERVIEWER NAMES:
1. ___________________________________

2. ___________________________________

SECTION A

1. **Location (nearest MRT): __________**

2. **Age (in years): __________________**

3. **Gender:**
1) Male ☐ 2) Female ☐

4. **Ethnicity**:
1) Malay ☐ 2) Indian ☐ 3) Chinese ☐ 4) Others (please specify):

5. **Height:**
1) **________________ (cm) OR _________ (feet) ________ (inches)**
2) Declined to answer ☐ 3) Don't know ☐

6. **Weight:**
1) **________________ (kg) OR _____________ (lbs)**
2) Declined to answer ☐ 3) Don't know ☐

7. In a typical week, how many minutes do you spend doing moderate or vigorous activity?
1) 0 - 49 ☐ 2) 50 - 99 ☐ 3) 100 - 149 ☐ 4) ≥150 ☐

8. Do you have children who are 18 years old or younger?
0 ☐ 1 ☐ 2 ☐ 3 ☐ 4 ☐ ≥5 ☐

SECTION B – **Beverage Screener**

B.1

How often did you consume one serving of ______ in the past year?		**Frequency**					**One Serving Equivalent**
		Per Day	Per Week	Per Month	Never or Rarely		1 glass, 1 bottle, 1 pack, 1 cup
Item No.	***Type of Beverage***						
1	Fruit or vegetable juice (100% juice, no added sugar), freshly squeezed or packed						
2	Fruit or vegetable juice NOT 100% juice (include syrups or cordials with water added, fruit drinks, punches) e.g. Ribena, Minute Maid						
3	Sweetened canned or bottled tea e.g. lemon/peach tea, milk tea, jasmine green tea, chrysanthemum tea						
4	Tea brewed unsweetened or less sweet (green tea, herbal teas, black tea, oolong tea, teh siew dai)						
5	Tea brewed sweetened (teh tarik, teh-C, teh-O)						
6	Canned or bottled coffee e.g. latte, mocha						
7	Coffee brewed unsweetened or less sweet (Kopi-kosong, kopi-siew dai)						
8	Coffee brewed sweetened (kopi-o, Kopi-c, café latte)						
9	Other non-carbonated (non-fizzy) sweetened drinks e.g. sugarcane juice, water chestnut drink, barley drink						
10	Energy or Sport drinks (100 plus ®, Red bull ®, H-Two-O ®)						
11	Carbonated (fizzy) soft drinks, sugar -free e.g. Coke Zero ®, Diet Pepsi®						
12	Carbonated (fizzy) soft drinks, regular e.g. regular Coca-Cola ®, Fanta ®, Pepsi®						

13	Soya milk, low sugar, no added sugar or flavouring e.g. chocolate, fruit, red bean						
14	Soya milk NOT low sugar, or with flavouring						
15	Milkshake e.g. chocolate, strawberry, banana						
16	Yoghurt drinks e.g. Yakult ®, lassi						
17	Malted drinks (e.g. Milo ®)						
18	Water, plain (including bottled)						

INTERVIEWER'S GUIDE: Showcard #1

B-2

How do you typically consume beverages (excluding water)?

a. **With a meal such as lunch or dinner** ☐
b. **With snacks** ☐
c. **By itself** ☐

INTERVIEWER'S GUIDE: Showcard #2

B-3

From where do you generally buy beverages (excluding water)?

a. **Vending machines** ☐
b. **Supermarkets (e.g. Shen Siong, NTUC Fairprice)** ☐
c. **Convenience stores (e.g. 7-11)** ☐
d. **Hawker centres/food courts** ☐
e. **Cafes** ☐
f. **Restaurants** ☐
g. **Others (please specify)**______________________

SECTION C

Sugar-Sweetened Beverages (SSBs) definition:
(http://www.cdph.ca.gov/SiteCollectionDocuments/StratstoReduce_Sugar_Sweetened_Bevs.pdf)

Sugar-sweetened beverages are those that contain caloric sweeteners and include:

INTERVIEWER GUIDE: Showcard #3

- Carbonated (fizzy) soft drinks, regular e.g. regular Coca-Cola ®, Fanta ®, Pepsi®
- Fruit or vegetable juices which are NOT 100% juice (include syrups or cordials with water added, fruit drinks, punches and ades such as lemonade) e.g. Ribena, Minute Maid
- Energy or Sport drinks (e.g. 100 plus ®, Red bull ®, H-Two-O ®)
- Sweetened canned or bottled tea e.g. lemon/peach tea, milk tea, jasmine green tea, chrysanthemum tea
- Tea brewed sweetened (e.g. Teh Tarik, teh C, teh-O, green tea frappacuccino)
- Coffee brewed sweetened (e.g. kopi-o, Kopi-c, café latte)
- Canned or bottled coffee e.g. latte, mocha
- Other non-carbonated (non-fizzy) sweetened drinks e.g. sugarcane juice, water chestnut drink, barley drink
- Soyamilk NOT low sugar, including flavoured
- Milkshake e.g. chocolate, strawberry, banana
- Malted drinks (e.g. Milo ®)
- Yoghurt drinks e.g. Yakult ®, lassi

In contrast, beverages which are not sweet include the following

INTERVIEWER GUIDE: Showcard #4

- Fruit or vegetable juice, 100% juice, NO added sugar
- Carbonated (fizzy) soft drinks, sugar free e.g. Diet Pepsi ®, Coke Zero®, Diet Fanta®, Sprite Zero®
- Tea brewed unsweetened or less sweet (green tea, herbal teas, black tea, oolong tea)
- Coffee brewed unsweetened
- Soya milk, no sugar added
- Cow's milk, no added sugar
- Water

1. In your opinion, does drinking large amounts of sugar sweetened beverages lead to health problems?

1) Yes ☐ 2) No ☐ 3) Don't know ☐

2. If yes, which health problem(s) would drinking large amounts of sugar sweetened beverages lead to?

1) ☐ **Overweight or obesity**

2) ☐ **Diabetes**

3) ☐ **Cancer**
4) ☐ **Heart disease**
5) ☐ **Poor dental health**
6) ☐ **Others: please specify** ______________________________
7) ☐ **None**
8) ☐ **Don’t know**

SECTION D

1. How much responsibility do you think each of the following groups has for **causing** the current obesity situation in Singapore?

INTERVIEWER'S GUIDE: Showcard #5<br>Please indicate 1 tick for each row as per participant's response

No.		No responsibility (1)	Little responsibility (2)	Moderate Responsibility (3)	Large Responsibility (4)	Very large responsibility (5)
a	People themselves					
b	Family members					
c	Doctors or health care professionals					
d	Food industry					
e	School					
f	Government policies					
g	Employers					

(Modified from The Associated Press-NORC Center for Public Affairs Research, Perceptions of obesity)

2. How much responsibility do you think each of the following groups has for **solving** the country's current obesity situation?

INTERVIEWER'S GUIDE: Showcard #5
Please indicate 1 tick for each row as per participant's response

No.		No responsibility (1)	Little responsibility (2)	Moderate Responsibility (3)	Large Responsibility (4)	Very large responsibility (5)
a	People themselves					
b	Family members					
c	Doctors or health care professionals					
d	Food industry					
e	School					
f	Government policies					
g	Employers					

(Modified from The Associated Press-NORC Center for Public Affairs Research, Perceptions of obesity)

SECTION E

1. ***Policy 1 – Labelling***

**INTERVIEWER GUIDE:** Showcard #6

a. Consider a policy in which, all beverages will be labelled with stickers. Beverages which have high amounts of sugar will be labelled with a red sticker, those with a moderate amount of sugar will be labelled with an orange sticker, and those with low amounts of sugar will be labelled with a green sticker. This will help consumers identify heathier and less healthy beverages based on their sugar content.

INTERVIEWER GUIDE: Showcard #7 – Scale

1. I support this policy

Strongly agree	Agree	Neither agree nor disagree	Disagree	Strongly disagree

2. I think that this policy would help reduce the consumption of SSBs in Singapore in general

Strongly agree	Agree	Neither agree nor disagree	Disagree	Strongly disagree

3. I think that this policy would reduce my own consumption of SSBs

Strongly agree	Agree	Neither agree nor disagree	Disagree	Strongly disagree

4. What other thoughts do you have about this policy?

INTERVIEWER GUIDE: Showcard #8

b. Consider a policy in which beverages that have added sugars will have a warning label such as "Drinking beverages with added sugar(s) contributes to obesity, diabetes and tooth decay". Beverages that have no added sugars will not have this label.

INTERVIEWER GUIDE: Showcard #9 – Scale

1. I support this policy

Strongly agree	Agree	Neither agree nor disagree	Disagree	Strongly disagree

2. I think that this policy would help reduce the consumption of SSBs in Singapore in general

Strongly agree	Agree	Neither agree nor disagree	Disagree	Strongly disagree

3. I think that this policy would reduce my own consumption of SSBs

Strongly agree	Agree	Neither agree nor disagree	Disagree	Strongly disagree

4. What other thoughts do you have about this policy?

2. *Policy 2 – Sugar Tax*

INTERVIEWER GUIDE: Showcard #10

Consider a policy in which the government will impose a tax of 20% on sugar sweetened beverages. For instance, sugar sweetened beverages which currently cost consumers SGD1 will cost consumers SGD1.20 after this policy is carried out. However, beverages which contain no added sugars will not be taxed.

INTERVIEWER GUIDE: Showcard #11 -scale

1. I support this policy

Strongly agree	Agree	Neither agree nor disagree	Disagree	Strongly disagree

2. I think that this policy would help reduce the consumption of SSBs in Singapore in general

Strongly agree	Agree	Neither agree nor disagree	Disagree	Strongly disagree

3. I think that this policy would reduce my own consumption of SSBs

Strongly agree	Agree	Neither agree nor disagree	Disagree	Strongly disagree

4. What other thoughts do you have about this policy?

3. Policy 3 - Limiting availability of unhealthy beverages and increasing availability of healthier beverages

INTERVIEWER GUIDE: Showcard #12

a. Consider a policy in which sugar sweetened beverages (SSBs) that contain high amounts of sugar will not be sold (either via retail or vending machines) within government institutions (eg. hospitals, schools, universities, government agencies etc.) Low sugar or unsweetened beverages will be sold.

INTERVIEWER GUIDE: Showcard #13- scale

1. I support this policy

Strongly agree	Agree	Neither agree nor disagree	Disagree	Strongly disagree

2. I think that this policy would help reduce the consumption of SSBs in Singapore in general

Strongly agree	Agree	Neither agree nor disagree	Disagree	Strongly disagree

3. I think that this policy would reduce my own consumption of SSBs

Strongly agree	Agree	Neither agree nor disagree	Disagree	Strongly disagree

4. What other thoughts do you have about this policy?

INTERVIEWER GUIDE: Showcard #14

b. Consider a policy in which sugar sweetened beverages (SSBs) which contain high amounts of sugar will not be sold (either via retail or vending machines) for a 300 meter (about 400 steps, 4 minute walk) radius around primary and secondary schools. Low sugar or unsweetened beverages will be sold.

INTERVIEWER GUIDE: Showcard #15-scale

1. I support this policy

Strongly agree	Agree	Neither agree nor disagree	Disagree	Strongly disagree

2. I think that this policy would help reduce the consumption of SSBs in Singapore in general

Strongly agree	Agree	Neither agree nor disagree	Disagree	Strongly disagree

3. I think that this policy would reduce my own consumption of SSBs

Strongly agree	Agree	Neither agree nor disagree	Disagree	Strongly disagree

4. What other thoughts do you have about this policy?

Policy 4 - Access to Potable Drinking Water

INTERVIEWER GUIDE: Showcard #16

Consider a policy where all hawker centres and food courts will have water fountains installed so as to provide easy and free access to drinking water to the public.

INTERVIEWER GUIDE: Showcard #17 - scale

1. I support this policy

Strongly agree	Agree	Neither agree nor disagree	Disagree	Strongly disagree

2. I think that this policy would help reduce the consumption of SSBs in Singapore in general

Strongly agree	Agree	Neither agree nor disagree	Disagree	Strongly disagree

3. I think that this policy would reduce my own consumption of SSBs

Strongly agree	Agree	Neither agree nor disagree	Disagree	Strongly disagree

4. What other thoughts do you have about this policy?

Policy 5 - Accessibility of Healthier Beverage Alternatives

INTERVIEWER GUIDE: Showcard #18

Consider a policy in which no-sugar/low-sugar beverages options within government institutions will be made more easily available by requiring beverage vendors to provide more low or no sugar beverage options as compared to sugar sweetened beverage options, and by placing the low or no sugar beverages prominently for customers to see.

INTERVIEWER GUIDE: Showcard #19-scale

1. I support this policy

Strongly agree	Agree	Neither agree nor disagree	Disagree	Strongly disagree

2. I think that this policy would help reduce the consumption of SSBs in Singapore in general

Strongly agree	Agree	Neither agree nor disagree	Disagree	Strongly disagree

3. I think that this policy would reduce my own consumption of SSBs

Strongly agree	Agree	Neither agree nor disagree	Disagree	Strongly disagree

4. What other thoughts do you have about this policy?

Policy 6- Limiting Marketing

INTERVIEWER GUIDE: Showcard #20

a. Consider a policy in which any advertisements or marketing of sugar sweetened beverages (SSBs) across all media (e.g. television, billboards, banners, radio, magazines, apps, internet and direct marketing) will need to be accompanied by a prominent safety warning " Drinking beverages with added sugar(s) contributes to obesity, diabetes and tooth decay".

INTERVIEWER GUIDE: Showcard #21 -scale

1. I support this policy

Strongly agree	Agree	Neither agree nor disagree	Disagree	Strongly disagree

2. I think that this policy would help reduce the consumption of SSBs in Singapore in general

Strongly agree	Agree	Neither agree nor disagree	Disagree	Strongly disagree

3. I think that this policy would reduce my own consumption of SSBs

Strongly agree	Agree	Neither agree nor disagree	Disagree	Strongly disagree

4. What other thoughts do you have about this policy?

INTERVIEWER GUIDE: Showcard #22

b. Consider a policy in which advertisements of sugar sweetened beverages (SSBs) (e.g. posters, banners etc.) will be not be permitted within a 300 meter (about 400 steps) radius of primary and secondary schools.

INTERVIEWER GUIDE: Showcard #23-scale

1. I support this policy

Strongly agree	Agree	Neither agree nor disagree	Disagree	Strongly disagree

2. I think that this policy would help reduce the consumption of SSBs in Singapore in general

Strongly agree	Agree	Neither agree nor disagree	Disagree	Strongly disagree

3. I think that this policy would reduce my own consumption of SSBs

Strongly agree	Agree	Neither agree nor disagree	Disagree	Strongly disagree

4. What other thoughts do you have about this policy?

Policy 7- Limiting portion size

INTERVIEWER GUIDE: Showcard 24

d. **Consider a policy in which a limit is placed on the size of sugar sweetened beverages that can be sold at food service outlets. For instance restaurants, food courts, hawker centres, cafés, and convenience stores cannot sell sugar sweetened beverages at a size that exceeds 300 ml (about 1 standard cup or glass).**

INTERVIEWER GUIDE: Showcard 25 – Scale

1. I support this policy

Strongly agree	Agree	Neither agree nor disagree	Disagree	Strongly disagree

2. I think that this policy would help reduce the consumption of SSBs in Singapore in general

Strongly agree	Agree	Neither agree nor disagree	Disagree	Strongly disagree

3. I think that this policy would reduce my own consumption of SSBs

Strongly agree	Agree	Neither agree nor disagree	Disagree	Strongly disagree

4. What other thoughts do you have about this policy?

<u>SECTION F</u>

a) Diabetes is a condition of insufficient insulin.	True	False	Unsure
b) Diabetes is curable.	True	False	Unsure
c) Obesity does not increase the risk of having diabetes.	True	False	Unsure
d) Frequent urination is a symptom of diabetes.	True	False	Unsure
e) Diabetic patients do not need to go for regular eye check-up.	True	False	Unsure

<u>SECTION G</u>

1. **Over the past year, what is your average total household income (SGD) PER MONTH?**

    1) Below 2000 per month ☐
    2) 2000-3999 per month ☐
    3) 4000-5999 per month ☐
    4) 6000 – 9999 per month ☐
    5) 10,000 and above ☐
    6) Declined to answer ☐
    7) Don't know ☐

2. **Which of the following best describes your <u>usual work status</u> over the <u>last 12 months</u>?**

    1) Working ☐
    2) Student (full-time) ☐
    3) Homemaker/Housewife ☐
    4) Retired ☐
    5) Unemployed (able to work) ☐
    6) Unemployed (unable to work)☐
    7) Others, please specify
    8) Declined to answer ☐

3. **What type of house do you live in?**

    1) HDB 1 – 2 room flat ☐
    2) HDB 3 room flat ☐
    3) HDB 4 room flat ☐
    4) HDB 5 room or executive flat ☐
    5) Others, please specify:
    6) Declined to answer ☐
    7) Don’t know ☐

4. **What is your highest level of education attained?**

    1) No formal education/Primary ☐
    2) PSLE ☐
    3) Secondary ☐
    4) O’/N Level or NTC-3 certificate or its equivalent ☐
    5) ‘A’ Level or NTC 1-2 certificate in office or business skills or its equivalent ☐
    6) Polytechinic diploma ☐
    7) Other diploma & professional ☐
    8) University and above ☐
    9) Others (specify)
    10) Refused to answer ☐

5. **Have you been diagnosed as having a chronic medical condition by a doctor (Western-trained)?**

    1) Yes ☐
    2) No ☐

6. **If yes, please specify the condition:**

____

<u>SECTION H</u>

DATA QUALITY (To be filled by the interviewer, post-interview)

1. **Did other people apart from the main participant answer any questions, or in your opinion influence the answers given by the interviewer?**
   1) Yes ☐ 2) No ☐

2. **Overall, you would say the quality of the interview was**
   1) Poor ☐ 2) Moderate ☐ 3) Good ☐

If poor, please state why:
