# Supplementary material for: Perspectives of Adult Singaporeans toward Potential Policies to Reduce the Consumption of Sugar Sweetened Beverages—A Cross-Sectional Study
Source: Nutrients. 2021 Nov 25;13(12):4231. doi: 10.3390/nu13124231 (PMC8706143; doi:10.3390/nu13124231)
Supplement: Supplementary file 1 [file nutrients-13-04231-s001.zip › nutrients-1360538-supplementary/Supplementary Files/Supplementary Tables.pdf]

Perspectives of Adult Singaporeans toward Potential Policies to Reduce the Consumption of Sugar Sweetened Beverages — A Cross-Sectional Study

Supplementary Table S1: Description of policy scenarios presented to participants

**Policy**	**Description**
Labelling:	
1a. SSB traffic light labels	Coloured-coded labels corresponding to sugar levels<br>*"Consider a policy in which, all beverages will be labelled with stickers. Beverages which have high amounts of sugar will be labelled with a red sticker, those with a moderate amount of sugar will be labelled with an orange sticker, and those with low amounts of sugar will be labelled with a green sticker. This will help consumers identify heathier and less healthy beverages based on their sugar content"*
1b. SSB health warning labels	Warning labels on all beverages with added sugars<br>*"Consider a policy in which beverages that have added sugars will have a warning label such as "Drinking beverages with added sugar(s) contributes to obesity, diabetes and tooth decay". Beverages that have no added sugars will not have this label"*
2. SSB tax	Imposition of a 20% tax on all beverages with added sugars<br>*"Consider a policy in which the government will impose a tax of 20% on sugar sweetened beverages. For instance, sugar sweetened beverages which currently cost consumers SGD1 will cost consumers SGD1.20 after this policy is carried out. However, beverages which contain no added sugars will not be taxed>"*

Prohibiting of sales of SSB:	
3a. In government institutions	Prohibition of sales of SSB within all government institutions where only low sugar or unsweetened beverages are permitted to be sold. ***Consider a policy in which sugar sweetened beverages (SSBs) that contain high amounts of sugar will not be sold (either via retail or vending machines) within government institutions (eg. hospitals, schools, universities, government agencies etc.) Low sugar or unsweetened beverages will be sold***
3b. Around schools	Prohibition of sales of SSB within a 300m radius of all primary and secondary schools where only low sugar or unsweetened beverages are permitted to be sold. ***"Consider a policy in which sugar sweetened beverages (SSBs) which contain high amounts of sugar will not be sold (either via retail or vending machines) for a 300 meter (about 400 steps, 4 minute walk) radius around primary and secondary schools. Low sugar or unsweetened beverages will be sold."***
4. Increasing access to potable water in hawker centres	Installation of water fountains in all hawker centres and food courts. ***"Consider a policy where all hawker centres and food courts will have water fountains installed so as to provide easy and free access to drinking water to the public."***
5. Reduced visibility of SSB at government-owned institutions	Beverage vendors are required to display low or unsweetened beverages more prominently as compared to SSB. ***"Consider a policy in which no-sugar/low-sugar beverages options within government institutions will be made more easily available by requiring beverage vendors to provide more low or no sugar beverage options as compared to sugar sweetened beverage options, and by placing the low or no sugar beverages prominently for customers to see"***
SSB advertisement:	

6a. Warning labels in TV advertisement<br><br><br><br><br><br><br><br><br>6b. Restriction around schools	Any advertisement of SSB across all media will be required to display a prominent safety warning<br>***Consider a policy in which any advertisements or marketing of sugar sweetened beverages (SSBs) across all media (e.g. television, billboards, banners, radio, magazines, apps, internet and direct marketing) will need to be accompanied by a prominent safety warning "Drinking beverages with added sugar(s) contributes to obesity, diabetes and tooth decay".***<br><br>Prohibition of SSB advertisements within a 300m radius of all primary and secondary schools<br>***"Consider a policy in which advertisements of sugar sweetened beverages (SSBs) (e.g. posters, banners etc.) will be not be permitted within a 300 meter (about 400 steps) radius of primary and secondary schools."***
7. SSB portion size restriction	Maximum portion size of SSB sold at food service outlets limited to 300 ml.<br>***"Consider a policy in which a limit is placed on the size of sugar sweetened beverages that can be sold at food service outlets. For instance restaurants, food courts, hawker centres, cafés, and convenience stores cannot sell sugar sweetened beverages at a size that exceeds 300 ml (about 1 standard cup or glass"***

Supplementary Table S2: Determinants of support for less restrictive policies, odds ratios (95% CI).

	Product labelling		Built environment	Marketing	Choice architecture
	Traffic light labelling	Warning labels	Installing water fountains at eateries	Safety warning on SSB marketing	Reduced visibility of SSB at government-owned institutions
Demographic characteristics					
**Age (years)**					
21-40	1	1	1	1	1
41-64	1.23 (0.76 – 2.00)	1.34 (0.92 – 1.95)	0.84 (0.56 – 1.27)	1.15 (0.80 – 1.65)	1.00 (0.70 – 1.41)
≥65	0.99 (0.60 – 1.63)	1.37 (0.91 – 2.05)	0.86 (0.55 – 1.33)	1.09 (0.74 – 1.60)	1.11 (0.76 – 1.61)
**Gender**					
Male	1	1	1	1	1
Female	1.98* (1.32 – 2.97)	1.92* (1.39 – 2.64)	1.42* (1.01 – 1.99)	1.30 (0.95 – 1.76)	1.95* (1.45 – 2.63)
**Ethnicity**					
Chinese	1	1	1	1	1
Malay	1.29 (0.64 – 2.60)	0.98 (0.58 – 1.64)	0.96 (0.56 – 1.65)	0.76 (0.47 – 1.22)	0.76 (0.48 – 1.21)
Indian	0.99 (0.51 – 1.90)	0.91 (0.54 – 1.53)	1.90 (0.98 – 3.69)	1.32 (0.78 – 2.25)	1.67 (0.99 – 2.82)
**Other**[a]	-	1.55 (0.17 – 13.96)	-	1.98 (0.22 – 17.88)	1.00 (0.17 – 6.06)
**Have children ≤18 years old**					
No	1	1	1	1	1
Yes	1.31 (0.82 – 2.09)	1.15 (0.80 – 1.64)	0.87 (0.60 – 1.26)	1.16 (0.82 – 1.63)	1.21 (0.87 – 1.68)
**Housing unit**					
3-room	1	1	1	1	1
4-room	0.86 (0.53 – 1.37)	0.92 (0.63 – 1.34)	0.95 (0.64 – 1.42)	1.37 (0.96 – 1.95)	1.14 (0.81 – 1.61)
5-room	1.00 (0.56 – 1.79)	0.79 (0.50 – 1.23)	0.91 (0.56 – 1.47)	0.93 (0.61 – 1.42)	0.99 (0.65 – 1.49)
**Monthly household income (SGD)**					
<4000	1	1	1	1	1
4000-5999	1.08 (0.63 – 1.87)	0.76 (0.49 – 1.16)	1.56 (0.96 – 2.54)	0.67 (0.44 – 1.01)	0.94 (0.63 – 1.40)
≥6000	1.08 (0.64 – 1.84)	0.68 (0.45 – 1.03)	1.02 (0.66 – 1.57)	0.96 (0.64 – 1.46)	1.09 (0.73 – 1.61)
**Education**					
Primary	1	1	1	1	1
Secondary	1.43 (0.82 – 2.50)	1.05 (0.65 – 1.69)	1.54 (0.95 – 2.48)	0.79 (0.50 – 1.23)	0.96 (0.63 – 1.46)
Post-secondary	1.85 (0.93 – 3.65)	0.99 (0.58 – 1.69)	1.34 (0.78 – 2.29)	0.80 (0.48 – 1.32)	0.95 (0.59 – 1.54)
Tertiary	1.20 (0.69 – 2.07)	0.66 (0.41 – 1.04)	1.43 (0.88 – 2.30)	0.71 (0.45 – 1.12)	1.00 (0.65 – 1.54)
**Work status**					
Not employed	1	1	1	1	1
Employed	1.05 (0.69 – 1.59)	0.69 (0.50 – 0.97)	0.93 (0.65 – 1.32)	1.05 (0.76 – 1.44)	0.96 (0.71 – 1.31)
Student	1.04 (0.47 – 2.32)	1.17 (0.59 – 2.32)	1.30 (0.63 – 2.68)	1.01 (0.55 – 1.85)	0.80 (0.45 – 1.41)

Health characteristics					
**BMI (kg/m²)**					
<23	1	1	1	1	1
23-<27.5	0.81 (0.51 – 1.29)	0.78 (0.54 – 1.13)	0.99 (0.67 – 1.46)	0.98 (0.69 – 1.39)	0.74 (0.53 – 1.04)
≥ 27.5	1.20 (0.61 – 2.34)	0.69 (0.43– 1.11)	1.34 (0.77 – 2.33)	0.88 (0.56 – 1.39)	0.89 (0.57 – 1.40)
**Exercise**					
<150min/week	1	1	1	1	1
≥150min/week	1.03 (0.66 – 1.60)	0.65* (0.46 – 0.91)	1.15 (0.79 – 1.69)	0.97 (0.69 – 1.35)	0.85 (0.62 – 1.17)
**Chronic medical conditions**					
No	1	1	1	1	1
Yes	1.32 (0.85 – 2.04)	1.62* (1.14 – 2.29)	1.01 (0.71 – 1.45)	1.08 (0.78 – 1.49)	1.12 (0.82 – 1.53)
**Consumption of SSB**					
Non-daily	1	1	1	1	1
Daily	1.48 (0.99 – 2.22)	0.99 (0.72 – 1.36)	0.82 (0.58 – 1.16)	1.08 (0.80 – 1.47)	0.85 (0.63 – 1.14)
Knowledge and Perceptions					
SSB cause health problems					
No/Unsure	1	1	1	1	1
Yes	1.35 (0.75 – 2.41)	0.99 (0.60 – 1.63)	1.44 (0.87 – 2.37)	1.29 (0.81 – 2.06)	2.34* (1.48 – 3.69)
Diabetes mellitus knowledge					
Poor knowledge	1	1	1	1	1
Good knowledge	1.18 (0.77 – 1.80)	1.36 (0.97 – 1.90)	1.34 (0.94 – 1.91)	1.13 (0.82 – 1.56)	1.50* (1.10 – 2.05)
**Perceived responsibility for solving obesity** [b]					
People themselves	2.39* (1.35 – 4.23)	1.74* (1.04 – 2.90)	1.53 (0.89 – 2.64)	1.42 (0.86 – 2.36)	1.35 (0.82– 2.23)
Family members	1.62* (1.08 – 2.42)	1.18 (0.86 – 1.62)	1.22 (0.87 – 1.72)	1.34 (0.99 – 1.82)	1.29 (0.96 – 1.73)
Health care professionals	1.20 (0.78 – 1.85)	1.58* (1.11 – 2.24)	1.14 (0.79 – 1.64)	1.13 (0.82 – 1.56)	1.28 (0.94 – 1.76)
Food industry	1.28 (0.84 – 1.94)	0.93 (0.68 – 1.29)	1.20 (0.84 – 1.70)	1.42* (1.03 – 1.94)	1.31 (0.97 – 1.76)
School	1.48 (0.98 – 2.24)	0.92 (0.67 – 1.27)	1.10 (0.78 – 1.55)	1.39* (1.02 – 1.90)	1.50* (1.11 – 2.01)
Government policies	1.38 (0.91 – 2.09)	1.05 (0.76 – 1.45)	1.46* (1.03 – 2.07)	1.19 (0.87 – 1.62)	1.12 (0.84 – 1.51)
Employers	1.19 (0.64 – 2.22)	0.94 (0.59 – 1.49)	1.12 (0.67 – 1.88)	1.87* (1.13– 3.08)	1.44 (0.92 – 2.26)

BMI = body mass index; CI = confidence interval; SSB = sugar-sweetened beverages. *p-value<0.05 based on univariate logistic regression models. [a] Estimates were not generated due to low numbers (-). [b] Reference group comprises of participants who consider the stakeholder as having low-moderate responsibility in solving obesity.

Supplementary Table S3. Determinants of support for more restrictive policies, odds ratios (95% CI).

	Taxation	Restrictions			
	**SSB tax (20%)**	**Product availability at government-owned institutes**	**Product availability near schools**	**Advertising near schools**	**Portion sizes**
		Demographic characteristics			
**Age (years)**					
21-40	1	1	1	1	1
41-64	0.91 (0.64 – 1.28)	1.58* (1.06 – 2.35)	0.92 (0.64 – 1.32)	1.09 (0.76 – 1.58)	1.15 (0.81 – 1.64)
≥65	1.02 (0.70 – 1.47)	1.04 (0.70 – 1.56)	1.11 (0.75 – 1.63)	1.01 (0.68 – 1.49)	1.16 (0.79 – 1.70)
**Gender**					
Male	1	1	1	1	1
Female	1.35* (1.01 – 1.81)	1.54* (1.11 – 2.14)	1.59* (1.18 – 2.16)	2.48* (1.81 – 3.39)	1.69* (1.25 – 2.29)
**Ethnicity**					
Chinese	1	1	1	1	1
Malay	1.11 (0.69 – 1.77)	1.06 (0.62 – 1.80)	0.88 (0.55 – 1.42)	0.67 (0.42 – 1.08)	1.16 (0.71 – 1.88)
Indian	1.06 (0.66 – 1.72)	2.01 (1.06 – 3.82)	1.67 (0.97 – 2.88)	1.15 (0.68 – 1.94)	2.33* (1.31 – 4.14)
**Other**	-	0.56 (0.09 – 3.30)	2.19 (0.24 – 19.71)	-	-
**Have children ≤18 years old**					
No	1	1	1	1	1
Yes	1.01 (0.73 – 1.38)	1.50* (1.02 – 2.20)	1.51* (1.07 – 2.13)	1.42* (1.00 – 2.02)	0.92 (0.66 – 1.28)
**Housing unit**					
3-room	1	1	1	1	1
4-room	0.94 (0.67 – 1.31)	0.91 (0.62 – 1.33)	0.97 (0.68 – 1.38)	1.33 (0.93 – 1.89)	1.14 (0.80 – 1.61)
5-room	0.91 (0.60 – 1.37)	1.12 (0.70 – 1.80)	1.18 (0.77 – 1.82)	1.20 (0.78 – 1.84)	1.08 (0.71 – 1.64)
**Monthly household income (SGD)**					
<4000	1	1	1	1	1
4000-5999	0.90 (0.60 – 1.33)	0.79 (0.51 – 1.23)	1.01 (0.67 – 1.54)	0.94 (0.62 – 1.43)	1.17 (0.76 – 1.78)
≥6000	0.92 (0.63 – 1.35)	1.13 (0.72 – 1.76)	0.92 (0.61 – 1.37)	1.10 (0.73 – 1.67)	0.88 (0.59 – 1.31)
**Education**					
Primary	1	1	1	1	1
Secondary	1.53 (1.01 – 2.32)	0.90 (0.55 – 1.45)	1.13 (0.73 – 1.76)	1.06 (0.68 – 1.66)	1.44* (0.93 – 2.23)
Post-secondary	1.05 (0.66 – 1.68)	1.04 (0.59 – 1.81)	0.85 (0.52 – 1.39)	1.00 (0.61 – 1.66)	1.24 (0.76 – 2.03)
Tertiary	1.02 (0.67 – 1.55)	0.73 (0.45 – 1.18)	0.85 (0.55 – 1.31)	0.93 (0.59 – 1.45)	0.85 (0.55 – 1.30)
**Work status**					
Not employed	1	1	1	1	1
Employed	1.07 (0.80 – 1.45)	0.74 (0.52 – 1.04)	0.82 (0.59 – 1.12)	0.90 (0.65 – 1.24)	0.70 (0.51 – 0.95)
Student	0.56 (0.31 – 0.99)	0.45* (0.25 – 0.82)	0.57 (0.32 – 1.02)	0.49 (0.27 – 0.87)	0.66 (0.37 – 1.18)
Health characteristics					
**BMI (kg/m²)**					

<23	1	1	1	1	1
23 – < 27.5	0.73 (0.52 – 1.02)	1.01 (0.69 – 1.48)	0.68* (0.48 – 0.95)	0.86 (0.60 – 1.23)	0.98 (0.69 – 1.40)
≥ 27.5	1.04 (0.67 – 1.62)	1.49 (0.87 –2.56)	1.16 (0.72 – 1.88)	1.10 (0.68 – 1.79)	0.98 (0.62 – 1.55)
**Exercise**					
<150min/week	1	1	1	1	1
≥150min/week	1.01 (0.74 – 1.39)	1.04 (0.72 – 1.49)	0.65* (0.47 – 0.91)	1.07 (0.76 – 1.51)	1.03 (0.74 – 1.44)
**Chronic medical conditions**					
No	1	1	1	1	1
Yes	1.28 (0.95 – 1.74)	1.17 (0.82 – 1.65)	1.21 (0.88 – 1.67)	1.24 (0.89 – 1.72)	1.20 (0.87 – 1.65)
**Consumption of SSB**					
Non-daily	1	1	1	1	1
Daily	0.94 (0.71 – 1.26)	0.94 (0.68 – 1.31)	1.19 (0.88 – 1.61)	0.90 (0.66 – 1.22)	0.86 (0.64 – 1.16)
Knowledge and perceptions					
**SSB cause health problems**					
No/Unsure	1	1	1	1	1
Yes	0.97 (0.61 – 1.52)	1.28 (0.78 – 2.09)	1.08 (0.68 – 1.73)	1.38 (0.87 – 2.19)	1.35 (0.86 – 2.14)
**Diabetes mellitus knowledge**					
Poor knowledge	1	1	1	1	1
Good knowledge	1.20 (0.88 – 1.64)	1.73* (1.23 – 2.43)	1.50* (1.09 – 2.07)	1.75* (1.27 – 2.41)	1.60* (1.16 – 2.19)
**Perceived responsibility for solving obesity [b]**					
People themselves	1.21 (0.74 – 1.99)	0.93 (0.52 – 1. 65)	1.09 (0.65 – 1. 82)	1.00 (0.59 – 1. 69)	1.27 (0.76 – 2.10)
Family members	1.34* (1.00 – 1.79)	1.35 (0.98 – 1. 88)	1.35* (1.00 – 1. 83)	1.17 (0.86– 1. 59)	1.57* (1.16 – 2.12)
Health care professionals	1.06 (0.78 – 1.43)	1.34 (0.94 – 1. 91)	1.14 (0.83 – 1. 57)	1.46* (1.05 – 2.05)	1.43* (1.04 – 1. 98)
Food industry	1.06 (0.79 – 1.42)	1.14 (0.82 – 1. 60)	1.05 (0.77 – 1. 42)	1.11 (0.81 – 1. 52)	1.41* (1.03 – 1. 92)
School	1.14 (0.85– 1.52)	1.43* (1.02 – 2.00)	1.20 (0.89 – 1. 63)	1.40* (1.03 – 1. 92)	1.19 (0.88 – 1. 61)
Government policies	1.03 (0.77 – 1. 37)	1.11 (0.79 – 1. 54)	1.25 (0.92 – 1. 70)	1.06 (0.78 – 1. 44)	1.15 (0.85 – 1. 55)
Employers	0.98 (0.64 – 1. 49)	1.34 (0.81 – 2.24)	2.00* (1.21 – 3.29)	1.55 (0.95 – 2. 52)	1.06 (0.68– 1. 66)

BMI = body mass index; CI = confidence interval; SSB = sugar-sweetened beverages, *p-value<0.05 based on univariate logistic regression models. [a] Estimates were not generated due to low numbers (-). [b] Reference group comprises of participants who consider the stakeholder as having low-moderate responsibility in solving obesity.
